# Supplementary material for: Toward High-Performance Electrochromic Conjugated Polymers: Influence of Local Chemical Environment and Side-Chain Engineering
Source: Molecules. 2022 Dec 1;27(23):8424. doi: 10.3390/molecules27238424 (PMC9741092; doi:10.3390/molecules27238424)
Supplement: Supplementary file 1 [file molecules-27-08424-s001.zip › molecules-2048816-supplementary.pdf]

*Supplementary Materials*

# Toward High-Performance Electrochromic Conjugated Polymers: Influence of Local Chemical Environment and Side-Chain Engineering

## 1. Experimental section

Unless otherwise noted, all reagents were obtained from commercial sources and used without further purification. Scheme 1 shows the synthetic routes of T610FBTT810, DT6FBT, and DT48FBT. The detailed synthesis procedures are described as following:

**T610FBTT810**

T610FBTT810 was synthesised *via* Stille coupling reaction as described in Scheme 1. 5-fluoro-2,1,3-benzothiadiazole (1.0 g, 2.9 mmol) and tributyl[(4-(2-butyldecyl)thiophen-2-yl)]stannane (1.8 g, 2.9 mmol) were dissolved in dry toluene (20 mL) with $Pd(PPh_3)_4$ (65 mg) catalyst. The mixture was stirred under reflux condition for 24 h. The crude product was purified by column chromatography, obtaining a monobromine intermediate in 65% yield. The above monobromine intermediate (556 mg, 1 mmol), $Pd(PPh_3)_4$ (65 mg) and tributyl[(4-(2-hexyldecyl)thiophen-2-yl)]stannane (654 mg, 1 mmol) were mixed in dry toluene (20 mL), The mixture was stirred under reflux condition for 24 h. The crude product was subjected to silica gel column to give a brownish red powder. $^1H$ NMR (500 MHz, $CDCl_3$) δ: 8.07 (s, 1H), 7.95 (s, 1H), 7.74 (s, 1H), 7.12 (s, 1H), 7.06 (s, 1H), 2.65 (m, 4H), 1.71 (m, 2H), 1.27 (m, 60H), 0.88 (m, 12H). $^{13}C$ NMR (125 MHz, $CDCl_3$) δ: 159.87, 157.85, 153.57, 153.48, 149.80, 143.22, 142.14, 137.41, 137.39, 132.10, 132.04, 131.93, 131.89, 130.35, 125.93, 125.84, 123.84, 123.79, 123.59, 116.82, 116.56, 111.30, 111.18, 38.94, 38.91, 35.04, 34.91, 33.34, 31.93, 30.06, 30.04, 30.03, 29.71, 29.67, 29.37, 26.65, 26.64, 26.63, 22.69, 14.12. MS calculated for $C_{52}H_{83}FN_2S_3$, 850.57; found, 851.6.

**DT6FBT**

5-fluoro-2,1,3-benzothiadiazole (1.0 g, 2.9 mmol) and tributyl[(4-hexyl)thiophen-2-yl)]stannane (2.7 g, 6 mmol) were dissolved in dry toluene (50 mL) with $Pd(PPh_3)_4$ (130 mg) catalyst. The mixture was stirred under reflux condition for 24 h. The

crude product was purified by column chromatography, obtaining a brownish red powder in 70% yield. $^1H$ NMR (500 MHz, $CDCl_3$) δ: 8.07 (s, 1H), 7.92 (s, 1H), 7.64 (s, 1H), 7.12 (s, 1H), 7.05 (s, 1H), 2.72 (m, 4H), 1.73 (m, 4H), 1.38 (m, 12H), 0.94 (m, 6H). $^{13}C$ NMR (125 MHz, $CDCl_3$) δ: 159.73, 157.71, 153.33, 153.25, 149.58, 144.36, 143.34, 137.52, 137.50, 132.13, 132.09, 131.39, 131.32, 129.68, 125.67, 125.58, 122.84, 122.78, 122.57, 116.58, 116.32, 111.11, 110.99, 31.73, 31.70, 30.56, 30.52, 30.50, 30.41, 29.09, 29.06, 22.66, 22.65. MS calculated for $C_{26}H_{31}FN_2S_3$, 486.16; found, 486.6.

**DT48FBT**

5-fluoro-2,1,3-benzothiadiazole (1.0 g, 2.9 mmol) and tributyl[(4-(2-ethylhexyl)thiophen-2-yl)]stannane (3.4 g, 6 mmol) were dissolved in dry toluene (50 mL) with $Pd(PPh_3)_4$ (130 mg) catalyst. The mixture was stirred under reflux condition for 24 h. The crude product was purified by column chromatography, obtaining a brownish red powder in 70% yield. $^1H$ NMR (500 MHz, $CDCl_3$) δ: 8.07 (s, 1H), 7.96 (s, 1H), 7.74 (s, 1H), 7.12 (s, 1H), 7.06 (s, 1H), 2.66 (m, 4H), 1.72 (m, 2H), 1.33 (m, 40H), 0.88 (m, 12H). $^{13}C$ NMR (125 MHz, $CDCl_3$) δ: 159.86, 157.84, 153.55, 153.46, 149.79, 143.20, 142.11, 137.40, 137.38, 132.09, 132.03, 131.93, 131.89, 130.34, 125.91, 125.82, 123.84, 123.78, 123.59, 116.80, 116.54, 111.28, 111.16, 38.93, 38.92, 35.05, 34.88, 33.36, 33.34, 33.32, 33.03, 31.92, 30.04, 29.73, 29.72, 29.65, 29.37, 28.89, 26.62, 26.61, 23.09, 22.71, 22.69, 14.18, 14.12. MS calculated for $C_{42}H_{63}FN_2S_3$, 710.41; found, 710.9.

**2. Instruments and characterization**

$^1H$ and $^{13}C$ NMR spectra were tested on a Bruker AV-500 with tetramethylsilane (TMS) as an internal reference. MS was performed by using a Bruker Agilent1290/maXis impact. The geometry was optimized by density functional theory (DFT) calculations performed at the B3LYP/6-31G(d) level using the Gaussian 09. UV-vis spectra of the monomers dissolved in $CHCl_3$ were taken by using Perkin-Elmer Lambda 900 Ultraviolet-Visible Near-Infrared spectrophotometer. With an F-4500 fluorescence spectrophotometer (Hitachi), the fluorescence spectra of the monomers were determined. Scanning electron microscopy (SEM) images were obtained on a JEOL JSM-6700F scanning electron microscope.

All the electrochemical experiments and polymerization of monomers were performed in a one-compartment cell with the use of Model 263A potentiostat-galvanostat (EG&G Princeton

Applied Research) under computer control. For electrochemical tests, the working and counter electrodes were both Pt wires with a diameter of 1 mm, while the reference electrode (RE) was Ag/AgCl. The Ag/AgCl RE was prepared by chronoamperometry method at potential of 1.5 V for 100 s in hydrochloric acid (6 mol $L^{-1}$) and calibrated with the SCE system. $Bu_4NPF_6$ as electrolyte was dissolved in anhydrous $CH_2Cl_2$ (0.1 mol $L^{-1}$). All the solutions were distilled in a dry nitrogen stream before use. Polymer films were obtained by potentiodynamic regime. After polymerization, the films were washed repeatedly with anhydrous MeCN to remove the electrolyte and monomer.

Spectroelectrochemistry and kinetic studies of polymers were recorded on a Specord 200 plus (Analytik Jena) spectrophotometer and the potentials were controlled using Versa STAT 3 (Princeton Applied Research). The spectroelectrochemical cell consisted of a quartz cell, an Ag/AgCl electrode as reference electrode, a Pt wire as counter electrode, and an indium tin oxide (ITO) coated glass as the transparent working electrode. All measurements were carried out in MeCN containing $Bu_4NPF_6$ (0.1 mol $L^{-1}$).

The potentials were alternated between the reduced and oxidized states with a residence time of (2s, 5s) 10 s. The optical contrast at the specific wavelength ($\lambda$) was determined by $\Delta T$% values of polymer films, using the following equation:

$$\Delta T = |T_{ox} - T_{red}|$$

The colouration efficiency ($CE$) is defined as the relation between the injected/ejected charge as a function of electrode area ($Q_d$) and the change in optical density ($\Delta OD$) at the specific wavelength ($\lambda$) of the sample as illustrated by the following equation:

$$\Delta OD = \log(T_{ox}/\ T_{red})$$

$$CE = \Delta OD / Q_d$$

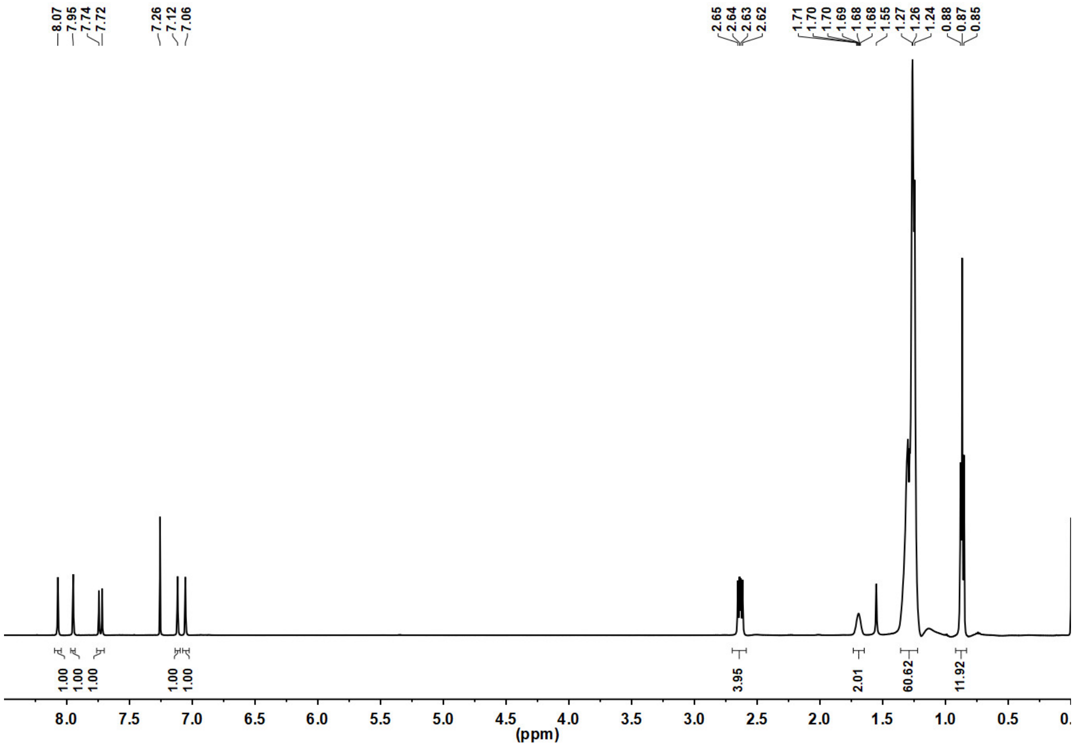


**Figure S1.** $^{1}H$ NMR spectrum of T610FBTT810.

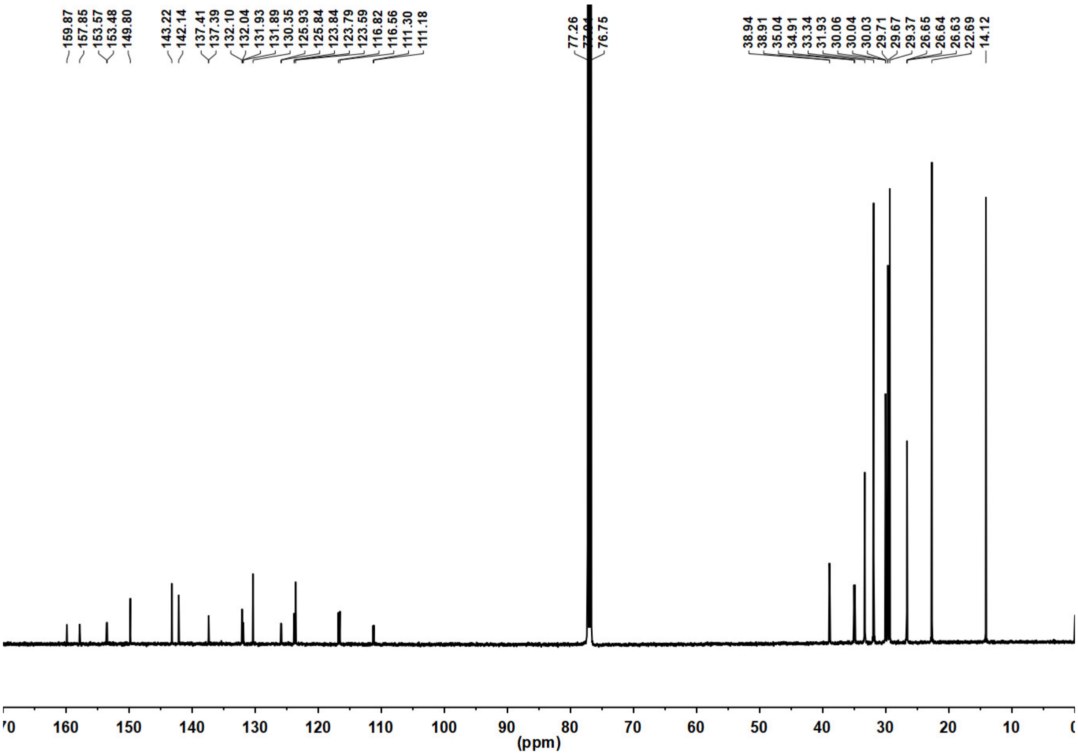


**Figure S2.** $^{13}C$ NMR spectrum of T610FBTT810.

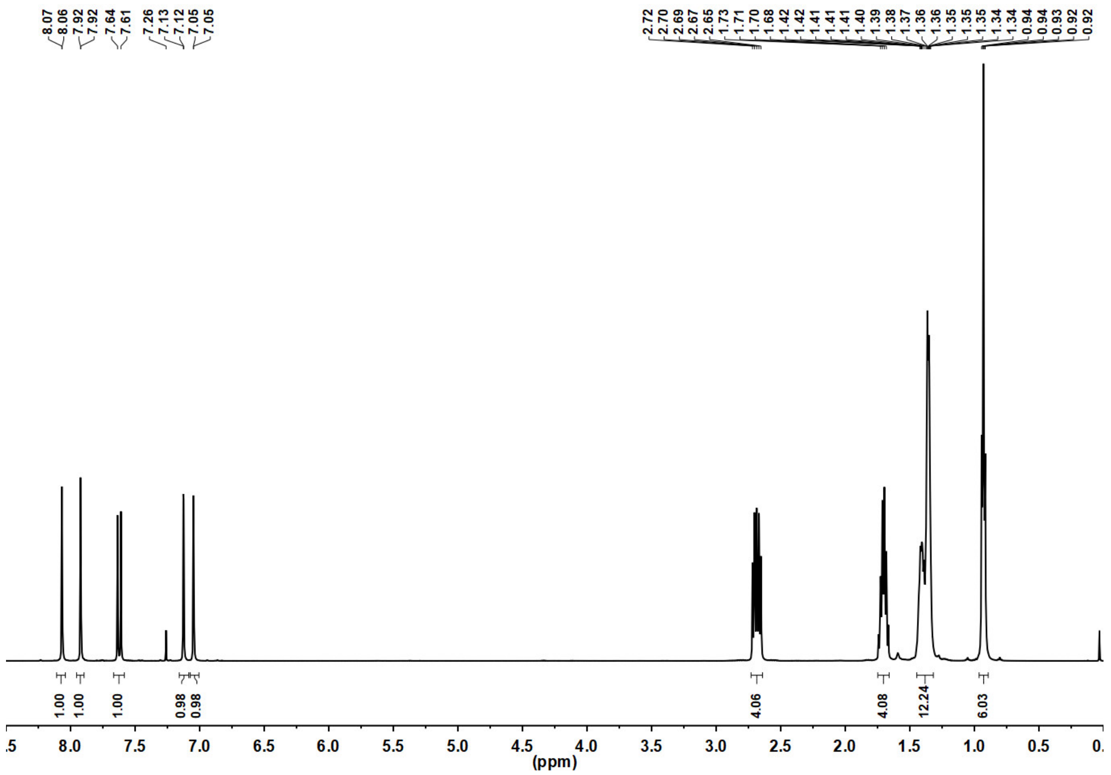

**Figure S3.** $^{1}H$ NMR spectrum of DT6FBT.

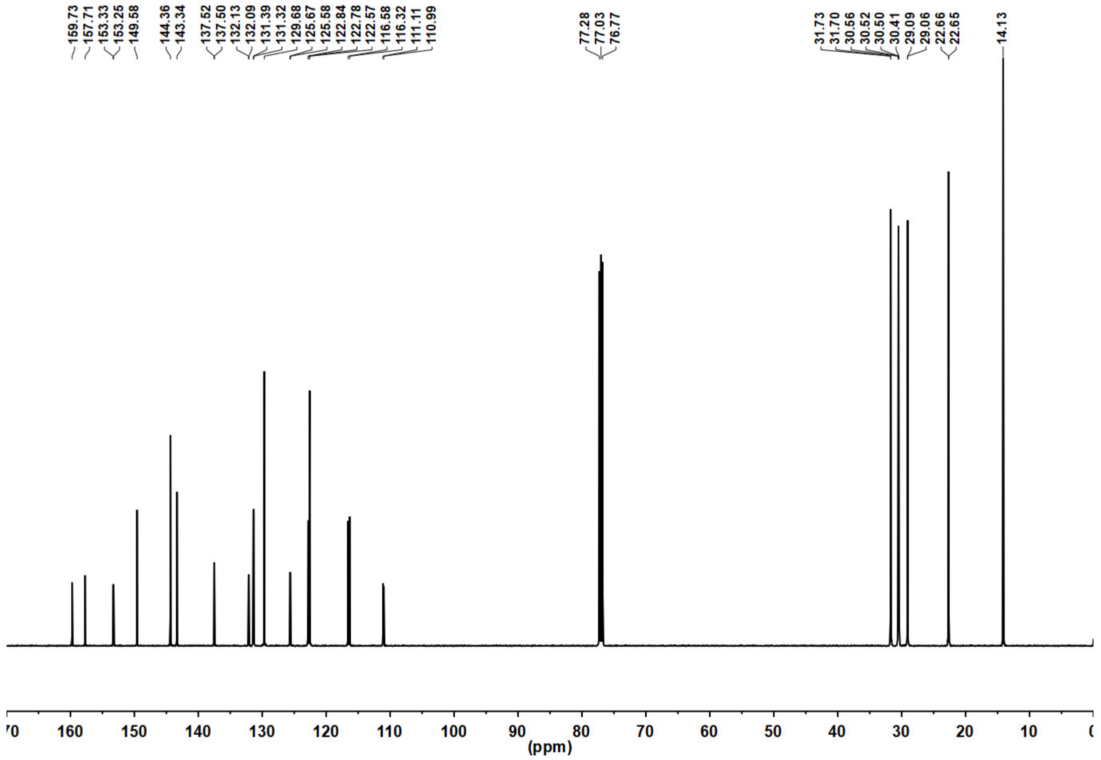

**Figure S4.** $^{13}C$ NMR spectrum of DT6FBT.

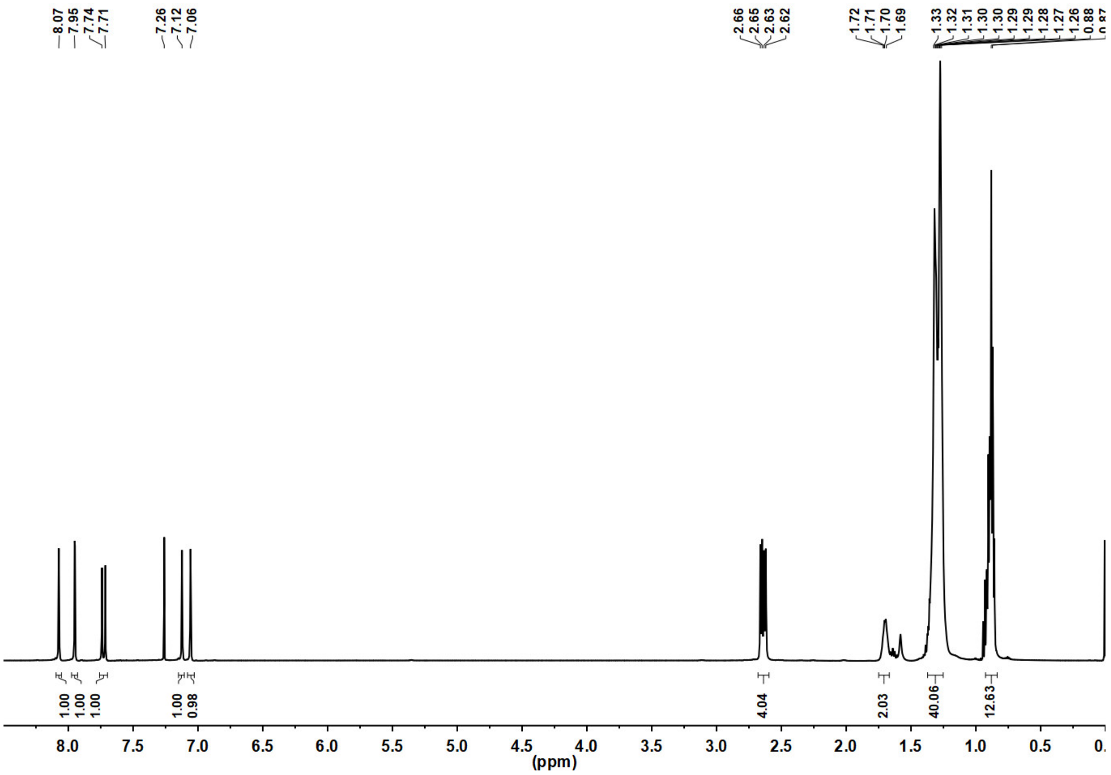


**Figure S5.** $^{1}H$ NMR spectrum of DT48FBT.

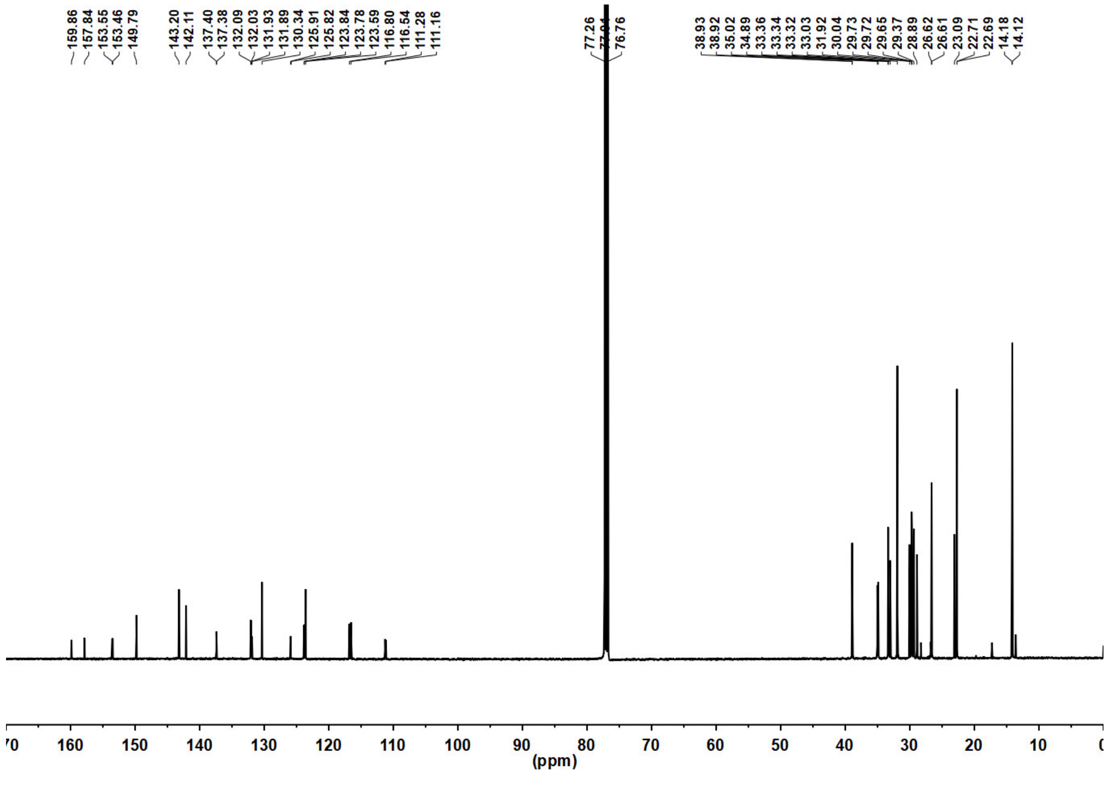


**Figure S6.** $^{13}C$ NMR spectrum of DT48FBT.

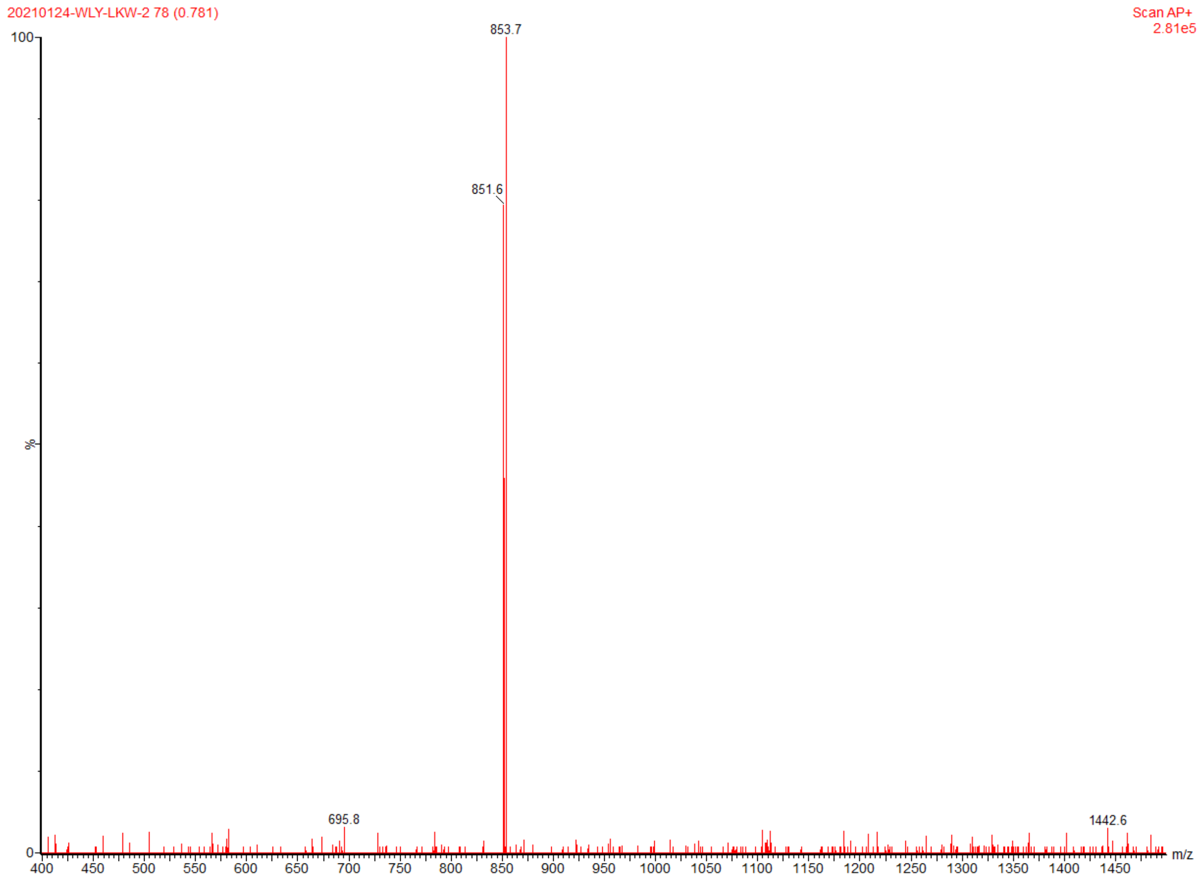


**Figure S7.** HRMS spectrum of T610FBTT810.

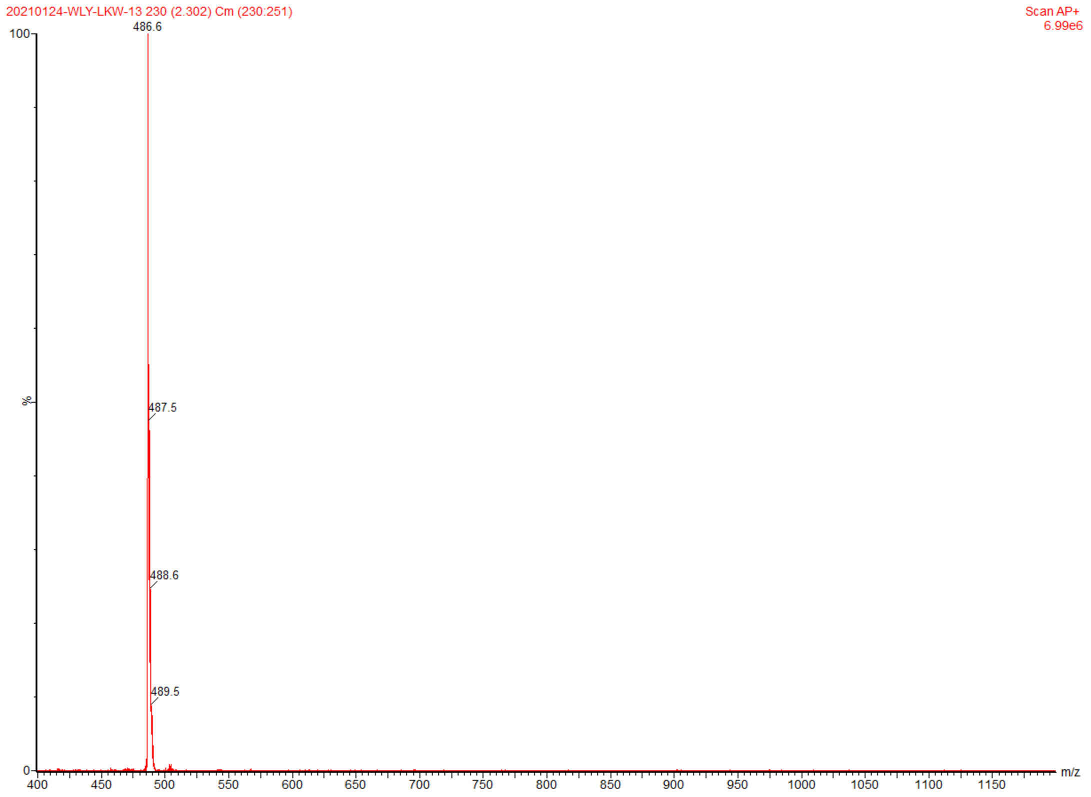


**Figure S8.** HRMS spectrum of DT6FBT.

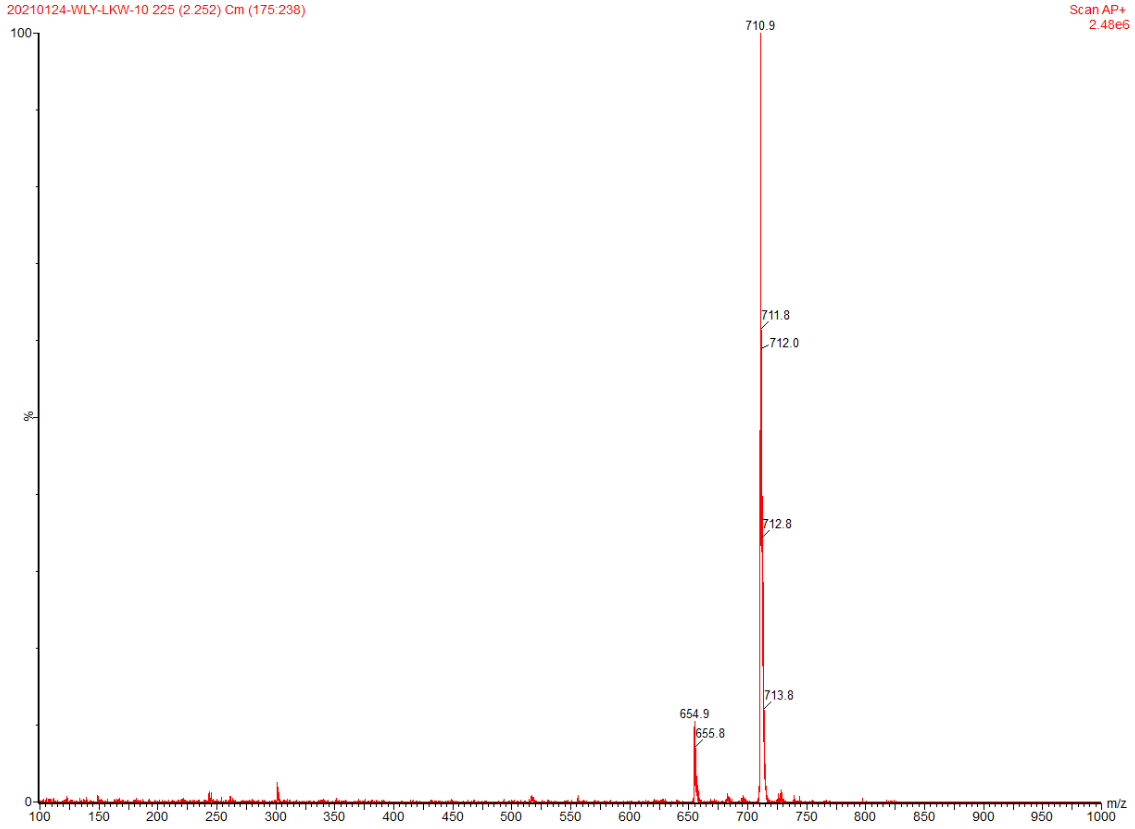


**Figure S9.** HRMS spectrum of DT48FBT.

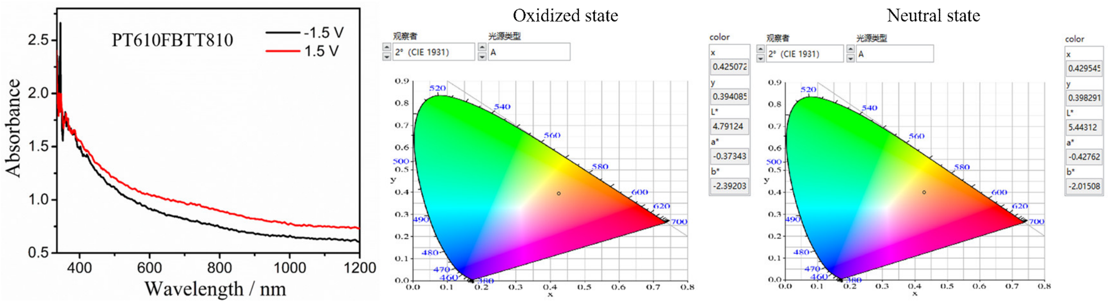


**Figure S10.** Spectroelectrochemistry of PT610FBTT810 on the ITO coated glasses in $CH_3CN$-$Bu_4NPF_6$ (A), chromaticity diagram (CIE 1931) of PT610FBTT810 in oxidized state (B) and in neutral state (C).

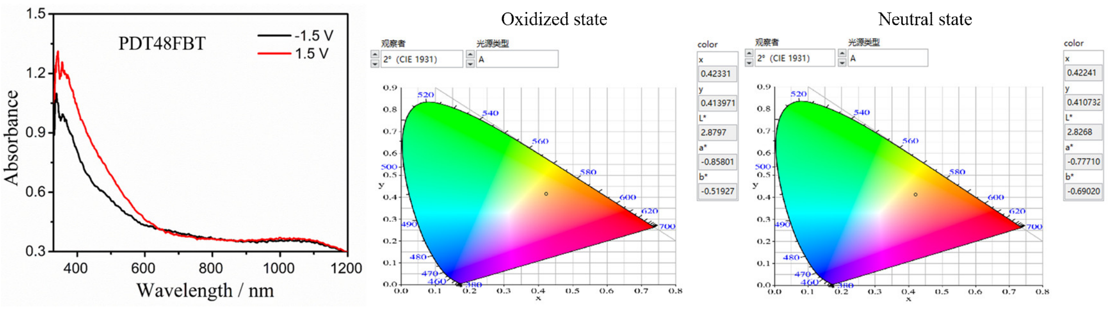


**Figure S11.** Spectroelectrochemistry of PDT48FBT on the ITO coated glasses in $CH_3CN$-$Bu_4NPF_6$ (A), chromaticity diagram (CIE 1931) of PDT48FBT in oxidized state (B) and in neutral state (C).

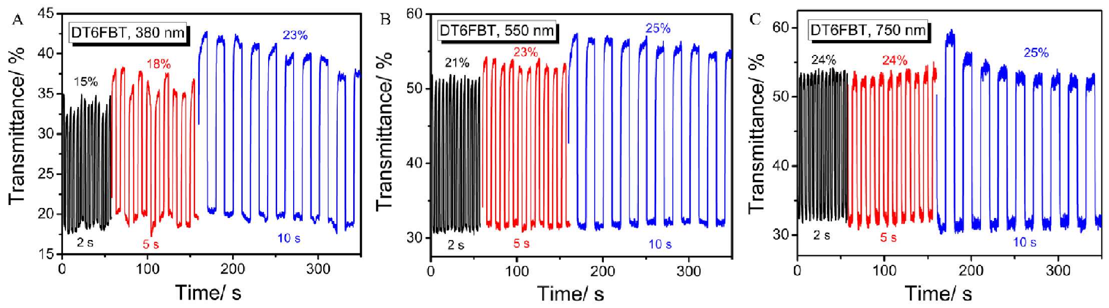


**Figure S12.** Time-transmittance curves of PDT6FBT at 380 nm with switching time of 2s, 5s, and 10 s (A); at 550 nm with switching time of 2s, 5s, and 10 s (B); at 750 nm with switching time of 2s, 5s, and 10 s (C) in monomer-free $CH_3CN$-$Bu_4NPF_6$ (0.1 mol $L^{-1}$).

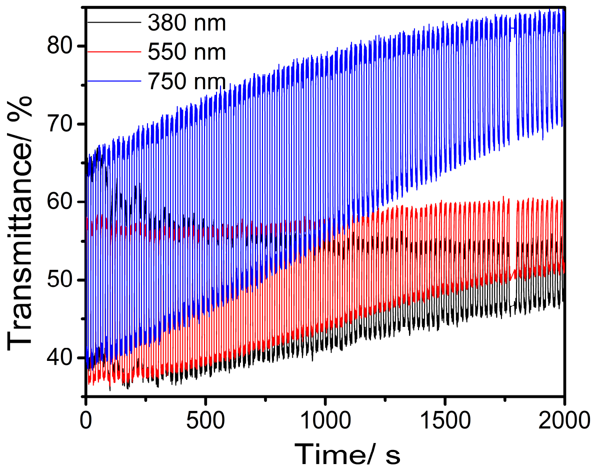


**Figure S13.** Kinetic stability of PDT6FBT at 380, 550, and 750 nm.
